# Gibberellin biosynthesis is required for CPPU-induced parthenocarpy in melon

**DOI:** 10.1093/hr/uhad084

**Published:** 2023-05-03

**Authors:** Yue Liu, Yang Li, Huixin Guo, Bingsheng Lv, Jing Feng, Huihui Wang, Zhonghua Zhang, Sen Chai

**Affiliations:** Engineering Laboratory of Genetic Improvement of Horticultural Crops of Shandong Province, College of Horticulture, Qingdao Agricultural University, Qingdao 266109, China; Engineering Laboratory of Genetic Improvement of Horticultural Crops of Shandong Province, College of Horticulture, Qingdao Agricultural University, Qingdao 266109, China; Engineering Laboratory of Genetic Improvement of Horticultural Crops of Shandong Province, College of Horticulture, Qingdao Agricultural University, Qingdao 266109, China; Engineering Laboratory of Genetic Improvement of Horticultural Crops of Shandong Province, College of Horticulture, Qingdao Agricultural University, Qingdao 266109, China; Engineering Laboratory of Genetic Improvement of Horticultural Crops of Shandong Province, College of Horticulture, Qingdao Agricultural University, Qingdao 266109, China; Engineering Laboratory of Genetic Improvement of Horticultural Crops of Shandong Province, College of Horticulture, Qingdao Agricultural University, Qingdao 266109, China; Engineering Laboratory of Genetic Improvement of Horticultural Crops of Shandong Province, College of Horticulture, Qingdao Agricultural University, Qingdao 266109, China

## Abstract

Spraying *N*-(2-chloro-4-pyridyl)-*N*′-phenylurea (CPPU), an exogenous cytokinin (CK) growth regulator, is the conventional method for inducing fruit set during melon (*Cucumis melo* L.) production; however, the mechanism by which CPPU induces fruit set is unclear. Through histological and morphological observations, fruit size was comparable between CPPU-induced fruits and normal pollinated fruits because CPPU-induced fruits had higher cell density but smaller cell size compared with normal pollinated fruits. CPPU promotes the accumulation of gibberellin (GA) and auxin and decreases the level of abscisic acid (ABA) during fruit set. Moreover, application of the GA inhibitor paclobutrazol (PAC) partially inhibits CPPU-induced fruit set. Transcriptome analysis revealed that CPPU-induced fruit set specifically induced the GA-related pathway, in which the key synthase encoding *gibberellin 20-oxidase 1* (*CmGA20ox1*) was specifically upregulated. Further study indicated that the *two-component response regulator 2* (*CmRR2*) of the cytokinin signaling pathway, which is highly expressed at fruit setting, positively regulates the expression of *CmGA20ox1*. Collectively, our study determined that CPPU-induced melon fruit set is dependent on GA biosynthesis, providing a theoretical basis for the creation of parthenocarpic melon germplasm.

## Introduction

Melon is an important cucurbit crop cultivated worldwide that is favored by consumers for its excellent flavor and nutritional value [1]. Yield and quality of melon are directly affected by the fruit set process, which is the starting point of fruit development and a prerequisite for fruit acquisition [2–5]. Compared with other horticultural crops, the fruit set of melon is highly sensitive to internal and external environmental factors during cultivation, which may cause fruit abortion. Melon fruit set typically occurs through either successful fertilization or parthenocarpy [5, 6]. The latter is an unpollinated fruit set mechanism that produces fruits without seeds and represents a desirable trait to obtain high-quality fruit under unfavorable environmental conditions [7]. Parthenogenetic fruit set is induced in naturally parthenogenetic varieties or by exogenous growth regulators. Due to a lack of parthenogenetic melon germplasm resources, spraying with *N*-(2-chloro-4-pyridyl)-*N*′-phenylurea (CPPU), an exogenous cytokinin (CK) growth regulator, is prevalent in melon production. CPPU application produces an excellent fruit set effect and better fruit yield and quality than natural pollination at a lower cost, and is thus a convenient and economic practice for producers [8, 9]. In recent years, a deterioration in fruit quality and food safety problems caused by misuse of CPPU have affected melon production [10]. Therefore, exploring the molecular mechanism of CPPU-induced fruit set may offer a solution for creating parthenogenetic melon germplasm with strong fruit set capacity.

Fruit set represents the beginning of fruit development and is accompanied by tissue changes and cell proliferation in the ovaries, which is regulated by many factors, such as sugar signals, water, and environmental factors [5, 7]. Previous studies have established that parthenogenetic fruit set is related to the coordinated action of different phytohormones [6, 7]. Parthenogenetic fruit species have much higher levels of some endogenous phytohormones, e.g. auxin, CK, and gibberellin (GA), than non-parthenogenetic fruit species [11, 12]. In plants, auxin was the first phytohormone determined to induce parthenogenesis. In horticultural crops such as strawberry, tomato, cucumber, and eggplant, exogenous application of auxin and its analogues or tissue-specific overexpression of *tryptophan 2-monooxygenase* (*iaaM*), catalyzing auxin biosynthesis, can enhance parthenogenetic fruit set capacity [12–14]. Genes involved in auxin transport and signaling pathway have also been shown to regulate parthenocarpic fruit set [15]. GA is another key phytohormone involved in parthenogenetic fruit set, and exogenous application of GA or its inhibitor affects fruit set [16]. Alterations in the expression of genes involved in GA biosynthesis and signaling pathways affect fruit set similarly to alterations in the expression of auxin-related genes [17, 18]. Both overexpression of *GA20ox* and silencing of *GA2ox* result in an increase of active GA in fruits and induce the production of parthenogenetic fruits [17, 18]. As crosstalk occurs between the auxin and GA biosynthetic pathways, exogenous application of either phytohormone or their analogues induces changes in the expression of biosynthetic genes and endogenous levels of the other phytohormone [14, 19]. In tomato, endogenous auxin and GA during fruit set induce *GA20ox1* and *GA3ox1* to activate GA biosynthesis, and *Gretchen hagen 3.2* to promote degradation of *Indoleacetic acid-induced protein 9* and *SlDELLA* and releasing *Auxin response factor 7* (*ARF7*), thereby inducing the completion of fruit set and later fruit development [20].

**Figure 1:**
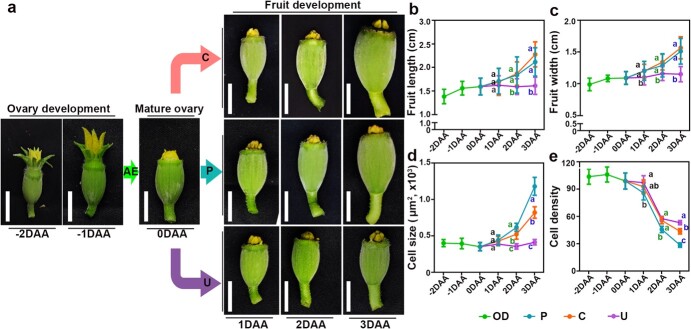
Morphological and cytological changes in melon during early ivf05 fruit development. **a** Morphological features during early development under different fruit set methods (−2 to 3 DAA). AE, artificial emasculation; OD, ovary development (green); C, CPPU-treated (orange); P, pollinated (blue); U, unpollinated (purple). Scale bar = 1 cm. **b**, **c** Length and diameter during early fruit development. Bars represent mean ± standard error of the mean (*n* = 10). **d**, **e** Size and density during early fruit pericarp development. Bars represent means of five fruits from five independent plants ± standard error of the mean. Comparing letters of the same color, significant differences (*P* < .05) were determined by one-way ANOVA and are indicated by different lowercase letters.

CK has a higher potential for use in horticultural crops than auxin or GA. In addition to being widely used in melon, CPPU, an analogue of CK, is also widely used in cucumber, tomato, kiwifruit, and other crops [9, 12, 21]. Transcription of genes involved in CK biosynthesis and signaling is enhanced in both CPPU-induced and naturally parthenocarpic fruits [12, 22]. In plants, *trans*-zeatin (tZ) is produced by adenosine phosphate-isopentenyl transferase, followed by cytochrome P450 monooxygenase (CYP735A) hydroxylating the prenyl side chain of isopentenyl adenosine phosphate [23]. In the non-parthenocarpic cucumber line 8419 s-1, transient expression of *CYP735A* induces the formation of parthenocarpic fruits [11]. CK signaling relies on a two-component signaling system. The catalytic receptor sensors histidine kinases (HKs) perceive the CK to be phosphorylated, and then the phosphate group is transferred by phosphotransmitters (AHP) to the key transcriptional factor type-B response regulator (RR) [24]. In pear, CPPU application induces the expression of *PbAHK4*, *PbAHK5-like*, *PbAHP5-like*, *PbRR9-like*, and *PbRR2-like*. *PbRR9-like* directly activates the expression of the auxin biosynthesis gene *indole-3-pyruvate monooxygenase 4* (*PbYUC4*) to increase the content of IAA (auxin) while directly inhibiting *9-cis-epoxycarotenoid dioxygenase 6* (*PbNCED6*) from the abscisic acid (ABA) biosynthesis pathway to reduce the content of ABA; fruit set in pears may not be dependent on the GA pathway [22]. Interestingly, in tomatoes and kiwifruit, CPPU-induced fruit set is partially dependent on GA and auxin biosynthesis; exogenous application of paclobutrazol (PAC) inhibits CPPU-induced parthenocarpy, but this can be rescued by exogenous GA application [21, 25]. The synthetic CK 6-benzyl adenine applied alone or in combination with 4-chlorophenoxyacetic acid significantly increases the levels of GA_1 + 3_ and GA_4 + 7_ and promotes GA biosynthesis genes in grape, resulting in the production of parthenogenetic fruit [26, 27]. Altogether, CPPU-induced fruit set relies on direct or indirect communication with other phytohormones, and the mechanisms of action vary among species. The mechanism by which CPPU induces melon fruit set remains to be elucidated.

In this study we induced melon fruit set through exogenous application of CPPU and studied changes at the histological, phytohormonal, and transcriptome levels. Ovaries with CPPU-induced and pollination-induced fruit set were significantly different from unpollinated ovaries from 1 day after anthesis (DAA). Although both CPPU treatment and pollination led to fruit set, their mechanisms were not identical. CPPU-induced melon fruit set was partially dependent on an increase in GA level, which might result from the direct induction of *GA20ox1* expression by *CmRR2*.

## Results

### Morphological and cytological features of melon fruit set

Fruit set is the beginning of fruit development. We observed the morphological and histological changes occurring in melon ovaries (fruit) that were unpollinated, artificially pollinated, or treated with CPPU. To determine the optimal dose of CPPU required to induce fruit set, mature ovaries were treated with CPPU solutions of 0, 2.5, 5, 10, 20, or 40 mg/l (Supplementary Data Fig. S1). As expected, fruit set ratio, fruit length, and fruit diameter were associated with CPPU concentration, reaching a plateau at ~10 mg/l (Supplementary Data Fig. S1b–d). Thus, 10 mg/l CPPU was used to spray ovaries. We then observed the morphological changes in melon ovaries (fruits) following different treatments at −2 to 4 DAA. Ovary length and width increased from −2 to −1 DAA, and growth rate then slowed down into the flowering period. After CPPU treatment or pollination, fruit size increased exponentially from anthesis to 3 DAA, but there was no significant difference between treatments. Interestingly, slow growth was sustained in unpollinated ovaries, which stopped at 1–2 DAA (Fig. 1a–c).

To analyze cytological changes during fruit set, we quantified pericarp cell density and cell size in all samples. Cell size and density did not differ significantly during ovary development. During fruit set, both pollinated and CPPU-induced fruits displayed larger cell size and considerably lower cell density than mature ovaries, whereas cell size remained the same but cell density declined in unpollinated ovaries (Fig. 1d and e). Paraffin sections revealed enlarged intercellular spaces and rupturing in unpollinated ovaries at 2 DAA (Supplementary Data Fig. S2). Interestingly, although pollinated and CPPU-induced fruits showed little difference in morphology during early fruit development, at the cellular level pollinated fruits displayed greater cell size and lower cell density than CPPU-induced fruits (Fig. 1d and e). This indicates that pollination and CPPU-induced fruit set are dependent on cell division and expansion, but there is a distinction between the two processes.

### Phytohormone levels during fruit set

**Figure 2:**
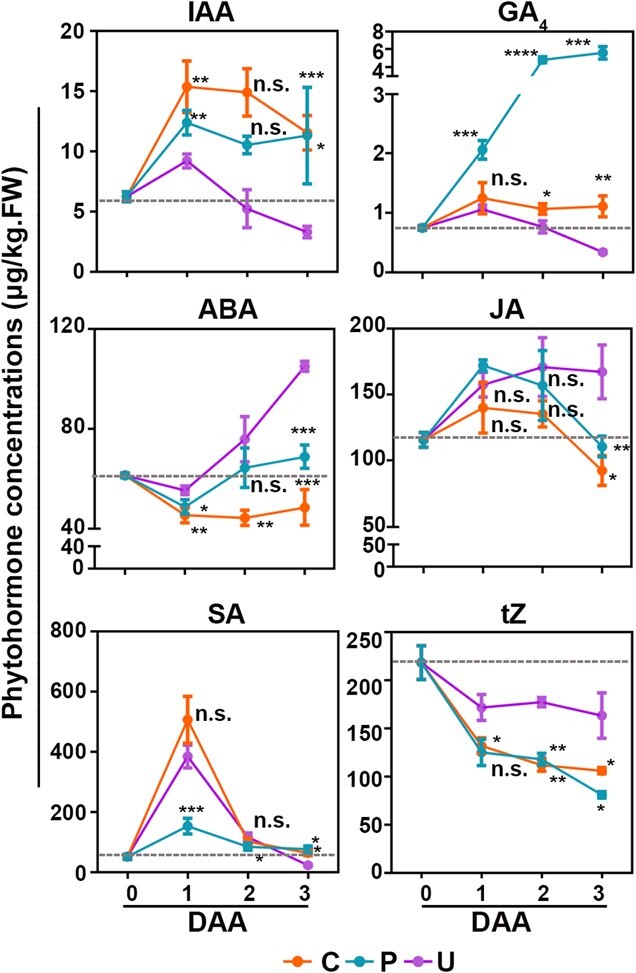
. Phytohormone levels of ivf05 during fruit set stages. Concentrations of the plant hormones IAA (auxin), GA_4_ (gibberellin), ABA (abscisic acid), JA (jasmonic acid), SA (salicylic acid), and tZ (cytokinin) were analyzed by LC–MS. Average phytohormone concentrations in early fruits under different fruit set methods were measured. C, CPPU-treated (orange); P, pollinated (blue); U, unpollinated (purple). The lines indicate the upward or downward trend of the hormone levels (dashed line, assigned a value for the initial hormone concentration of 0 DAA). Asterisks indicate significant differences compared with unpollinated fruit. n.s., not significant; ^*^P < .05; ^**^P < .01; ^***^P < .001; ^****^P < .0001 (one-tailed *t*-test). Error bars indicate mean ± standard deviation of at least three independent trials.

Fruit set requires changes in phytohormone levels and depends on cooperation among multiple phytohormones [7]. To analyze changes in phytohormone concentrations in melon fruits during fruit set, we determined the contents of endogenous phytohormones at multiple time points in fruits under different treatments (Fig. 2). Fruit set caused an increase in IAA content compared with fruits at 0 DAA, with a peak at 1 DAA in all treatments followed by a decrease at 2 DAA in unpollinated ovaries. CPPU treatment significantly inhibited accumulation of ABA in fruits, while the content of ABA in unpollinated ovaries increased significantly from 1 DAA. The content of GA_4_ was high in fruits and continued to increase in pollinated fruits but significantly reduced after 1 DAA in unpollinated ovaries, while other forms of GA showed irregular changes (Fig. 2, Supplementary Data Fig. S3), indicating that GA_4_ may play an important role during fruit set. CPPU treatment also caused an increase in GA_4_ content, but the extent of the increase was less than that in pollinated fruit. In terms of cytokinin, tZ content decreased in all samples following anthesis, and other forms of cytokinin varied dramatically (Fig. 2, Supplementary Data Fig. S3). Jasmonic acid levels increased in unpollinated fruit, with both fruit-set treatments inducing an increase followed by a decrease in jasmonic acid levels; salicylic acid levels varied dramatically among the three treatments (Fig. 2). In connection with the characteristics of early fruit developmental stages, contents of IAA, ABA, and GA were closely related to fruit set. Fruit set resulted in an increase in endogenous IAA and GA and a suppression of ABA, but there were some differences between pollination- and CPPU-induced fruit set.

**Figure 3:**
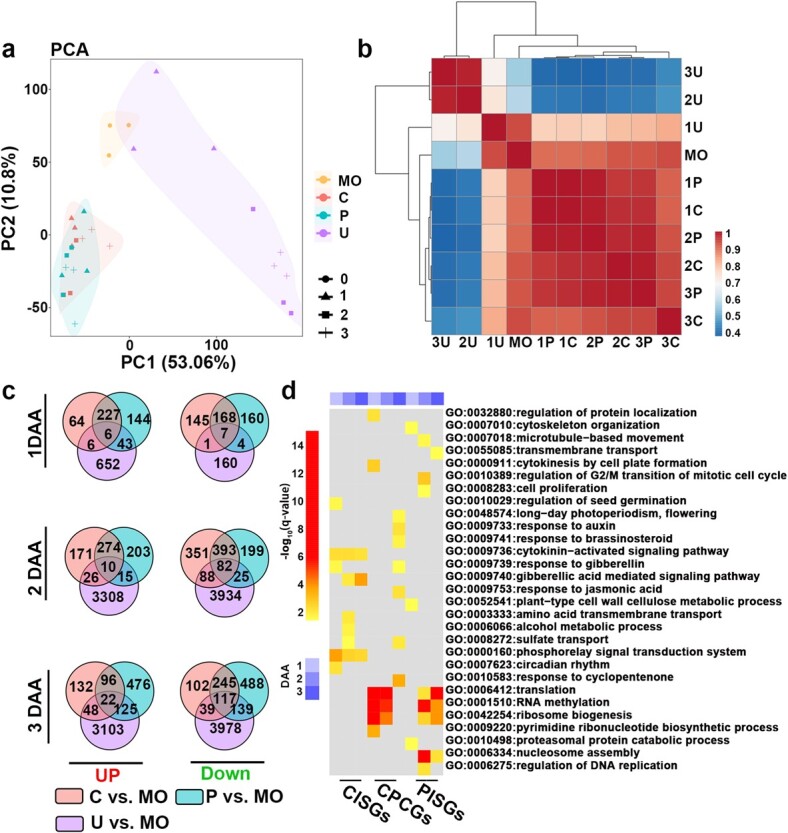
Transcriptome comparison among CPPU-treated, pollinated, and unpollinated fruits, and mature ovary. **a** PCA analysis of transcriptomes of all samples. MO, mature ovary; C, CPPU-treated; P, pollinated; U, unpollinated. **b** Heat map of the matrix of Pearson’s correlation coefficients based on gene expression. **c** Venn diagram of DEGs in the pairwise comparisons. **d** GO analysis of DEGs in fruit set. CISGs, CPPU-induced specific DEGs; PISGs, pollination-induced specific DEGs; CPCGs, CPPU- and pollination-induced common DEGs.

**Figure 4:**
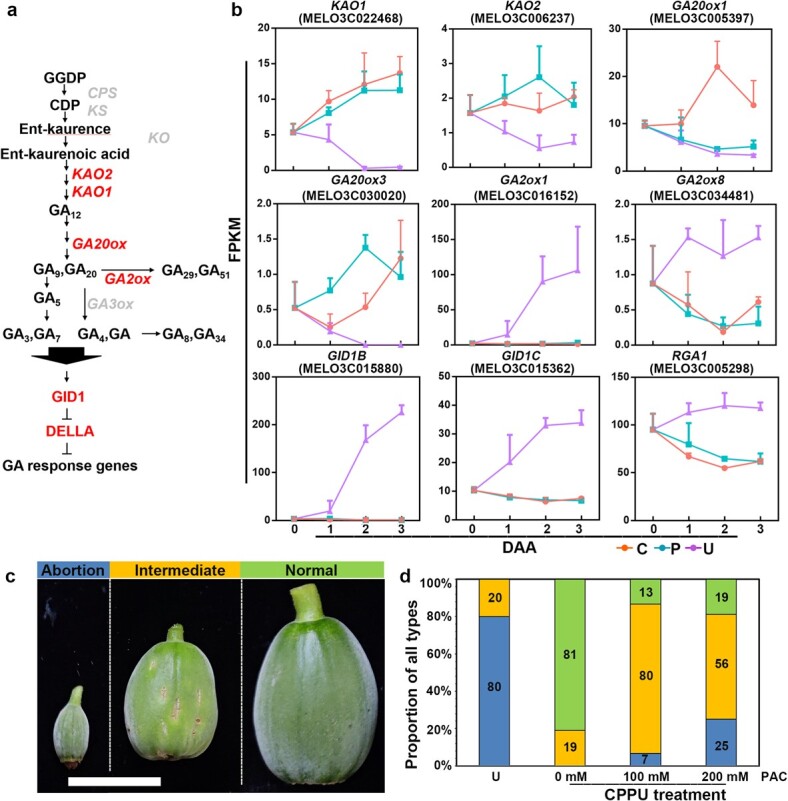
. The role of GA in CPPU-induced fruit set. **a** GA metabolism and signaling pathways. Red indicates genes with significant differences in expression under different treatments. **b** Transcript levels of GA-related genes under different treatments (0–3 DAA). C, CPPU-treated; P, pollinated; U, unpollinated. **c** Representative pictures of fruits after fruit set in different treatments. Fruits were collected 10 days after treatments. Bar = 2.5 cm. **d** Statistics of fruits after treatment with different fruit set methods. Different color blocks represent different degrees: blue, abortion fruit; yellow, intermediate state; green, normal fruit set.

### CPPU-induced fruit set occurs specifically through GA-related pathways

To study the mechanism of CPPU-induced fruit set, we performed transcriptome analyses in CPPU-induced, pollinated, and unpollinated fruits from anthesis to 3 DAA as well as mature ovaries. We mapped 150-bp paired-end reads to the melon genome at an average rate of 96% (Supplementary Data Table S1). The 30 samples had a similar distribution of number of fragments per kilobase per million reads (FPKM) (Supplementary Data Fig. S4). An average of 20 655 genes were expressed (FPKM >0) in these 30 samples. Based on developmental stage, principal component analyses (PCAs) clustered transcript profiles corresponding to tissues or cell types in the equatorial regions of the fruit. PCA of the 30 samples supported markedly different expression profiles in unpollinated fruits compared with CPPU-induced and pollinated fruits (Fig. 3a). A Pearson’s correlation coefficient heat map placed unpollinated fruits at 2 and 3 DAA in different clusters (Fig. 3b). We performed differential expression analysis on CPPU-induced, pollinated, and unpollinated fruits compared with mature ovaries at 1, 2, and 3 DAA. Differentially expressed genes (DEGs) were defined with adjusted *P*-values <.05 and absolute log_2_ (fold change) >1 (Supplementary Data Fig. S5), and we identified 12 184 DEGs within 1–3 DAA (Supplementary Data Fig. S6, Supplementary Data Table S2). Compared with CPPU-induced and pollinated fruits, unpollinated fruits had more DEGs from 2 DAA. To further explore the specific mechanism of CPPU-induced melon fruit set, we used Venn analysis to identify DEGs specific to the CPPU-induced fruit set (CISGs), DEGs specific to the pollination-induced fruit set (PISGs), and DEGs common to the CPPU- and pollination-induced fruit set (CPCGs) (Fig. 3c). We performed Gene Ontology (GO) enrichment analysis of DEGs at each study time point for each treatment (Supplementary Data Table S4). DEGs were significantly enriched in 46 biological processes during the fruit-set period. Besides genes associated with CK-related pathways (GO:0009736 and GO:0000160) co-enriched during the whole period after CPPU treatment, GA-related genes (GO:0009739 and GO:0009740) were specifically enriched at the early development stage, indicating a special role of GA in the fruit set induced by CPPU (Fig. 3d).

### CPPU-induced fruit set is partly dependent on gibberellin

GA is one of the key phytohormones in fruit set, and exogenous GA can induce fruit set in many plants [7]. Our detection of phytohormone concentrations and GO analysis implied that GA might function in CPPU-induced fruit set in melon, so we analyzed expression changes of GA-related genes. Compared with levels in mature ovules, expression of many GA metabolism genes was induced. Two genes encoding *ent*-kaurenoic acid hydroxylase (*KAO 1* and *2*) and the *GA20ox3* gene were significantly upregulated in CPPU-induced and pollinated ovaries but suppressed in unpollinated ovaries. *GA20ox1*, a key gene for inducing parthenocarpy, was specifically upregulated by CPPU treatment (Supplementary Data Fig. S12). Expression of two genes, *GA2ox1* and *GA2ox8*, encoding proteins involved in the inactivation of active gibberellase by 2β-hydroxylation, was suppressed during fruit set and enhanced in unpollinated ovaries. In addition, expression of two genes encoding GA receptors, *GID1B* and *GID1C*, and one gene encoding a DELLA protein, *RGA1*, was enhanced in unpollinated fruit but suppressed following fruit set (Fig. 4a and b). The fruit set process induced upregulation of active GA biosynthesis genes and downregulation of GA deactivation biosynthesis genes and signal transduction pathway genes, whereas the opposite trends were observed in unpollinated ovaries.

To study the relevance of GA in melon fruit set, mature ovaries were treated with different doses of GA_4 + 7_. Application of GA alone promoted fruit set in melon, which first increased and then decreased with increasing GA_4 + 7_ dose (Supplementary Data Fig. S7). The optimum dose was ~20 mg/l, but the fruit set rate was only 75%, which is lower than under CPPU treatment (Supplementary Data Fig. S7b). Furthermore, we inhibited GA biosynthesis in CPPU-treated ovaries by spraying PAC, a GA biosynthesis inhibitor, at 100 and 200 mm/l. The abortion rate increased from 0 to 25% with increasing concentrations of PAC compared with treatments with CPPU alone, indicating that CPPU-induced fruit set was partially suppressed (Fig. 4c and d). Interestingly, PAC application resulted in an increase in the proportion of fruits in an intermediate state in which fruit development was significantly slower despite fruit set. These results show that fruit set and fruit development of melon induced by CPPU are partly dependent on GA.

### CmRR2 modulates CPPU-induced fruit set by upregulating *CmGA20ox1* expression

**Figure 5:**
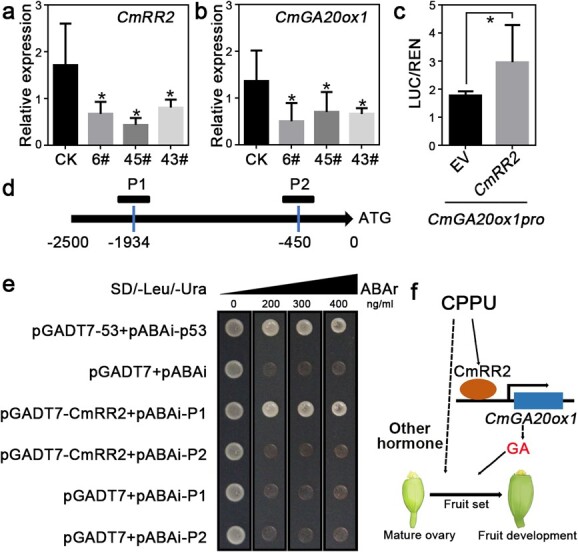
. Binding and stimulation of the *CmGA20ox1* promoter by *CmRR2*. **a**, **b** qRT–PCR analysis of *CmRR2* and *CmGA20ox1* in the fruits of wild-type and different *CmRR2* transgenic lines. **c** A dual luciferase assay was performed to validate the activation effect of *CmRR2* on the *CmGA20ox1* promoter. The ratio of the structural gene luciferase (LUC) to 35S-driven *Renilla* (REN) was calculated to represent the relative promoter activity. The empty vector (EV) was transfected as the control. Data are presented as mean ± standard deviation (*n* = 3, *t*-test: ^*^P < .05). **d** Schematic diagram of the promoter fragments of *CmGA20ox1* containing the promoter used for Y1H assay. **e** The Y1H assay revealed the interaction between CmRR2 and two pieces of DNA sequence (P1 and P2 indicated in **d**) from the *CmGA20ox1* promoter. **f** Model depicting the mechanism of melon parthenocarpy induced by CPPU.

CPPU has an underlying molecular mechanism similar to CK. Two-component RRs are essential in the CK signaling pathway, especially B-type RRs, which directly bind to the promoters of target genes to regulate gene expression. Transcriptome data revealed that *CmRR2* (MELO3C006693), the homolog of *AtRR2*, had the highest expression level among type-B *RR* genes at the fruit set stage and may play a key role in the signal transduction of CPPU-induced fruit set (Supplementary Data Fig. S8). To understand further the function of *CmRR2* in melon, we introduced the *CmRR2* coding sequence driven by the 35S promoter (*CmRR2*-OE) into the melon inbred line ivf05 through *Agrobacterium*-mediated cotyledon transformation and selected positive transplants using PCR analyses with primers targeting the vector. Three independent *CmRR2*-OE *T*_0_ transgenic lines were obtained. Surprisingly, the expression of *CmRR2* was downregulated in the *CmRR2*-OE lines (Fig. 5a). It is worth noting that other type-B *RR* genes were also repressed in the fruit (Supplementary Data Fig. S9), which suggested a possible co-repression of type-B *RR* members, including *CmRR2* itself, by ectopic overexpression of *CmRR2* [28, 44, 45]. CPPU-induced fruits showed altered expression of GA-related genes and further increased GA levels compared with mature ovaries and unpollinated fruits. As *CmGA20ox1* was specifically induced by CPPU treatment, we further explored the molecular mechanism of *CmGA20ox1* in CPPU-induced parthenocarpy. In *CmRR2* transgenic lines, qRT–PCR results showed downregulation of *CmGA20ox1* (MELO3C005397) and *CmKAO2* (MELO3C006237) (Fig. 5b, Supplementary Data Fig. S10). Further analysis showed that only the *CmGA20ox1* promoter had binding sites for RR2 at −450 and −1934 (Fig. 5b). Chromosome immunoprecipitation–DNA sequencing (ChiP-seq) of *Arabidopsis* RR10 revealed a distinct binding peak at the promoter of *Arabidopsis GA20ox1* (Supplementary Data Fig. S11). Dual luciferase assays showed that CmRR2 enhanced the *CmGA20ox1* promoter activity (Fig. 5c). Moreover, we proved CmRR2 binding to the *CmGA20ox1* promoter via a yeast one-hybrid (Y1H) assay (Fig. 5d and e). These results suggest that CmRR2 may directly activate *CmGA20ox1* expression.

## Discussion

Parthenocarpy is an ideal trait for agricultural production since it reduces labor costs and yields seedless fruits, which are popular among consumers. However, natural parthenocarpic germplasm resources are only available in a few crops, e.g. cucumber [7, 12]. Spraying growth regulators has become the predominant way of producing parthenogenetic fruits. CPPU is one of the main growth regulators for horticultural crops and has been widely used in tomato, cucumber, watermelon, peach, and other crops. Melon is one of the main cucurbit crops but lacks natural germplasm resources for parthenocarpy; therefore, its fruit set process is mainly induced by spraying CPPU [8, 9]. Nevertheless, the molecular mechanism by which CPPU induces fruit set in melon remains obscure.

In this study, we observed that after early rapid development the ovaries enter a plateau stage during anthesis. CPPU treatment and pollination cause an increase in the length and width of fruits, while morphological changes in unpollinated ovaries stop at 1–2 DAA (Fig. 1). Histological analysis revealed changes in cell density and size during fruit set, while unpollinated ovaries showed a cessation of cell growth, increased intercellular space, and even apoptosis at 2 DAA (Supplementary Data Fig. S2). This is consistent with the findings in pumpkin and cucumber, where ovary growth ceases at 1–2 DAA and fruits are morphologically and histologically distinguishable from those derived from unpollinated ovaries [11, 29]. This result was supported by transcriptome analysis. PCA clustering among different samples indicated convergent gene expression profiles among pollinated and CPPU-induced fruits, which were highly similar at 1–2 days after fruit set (Fig. 2). However, unpollinated ovaries at 2–3 DAA were significantly different from mature ovaries at both the histological and transcriptional levels. GO analysis of DEGs also showed that genes involved in cell proliferation and phytohormone-related pathways were enriched in developing fruits, while senescence- and dehydration-related genes were activated in unpollinated fruits (Supplementary Data Table S5). Our results indicate that transcriptional reprogramming during fruit set promotes cell division and expansion, and further fruit development. By contrast, unpollinated ovaries start the process of apoptosis from 1–2 DAA because they do not receive the signal for fruit set. Fruits produced through CPPU induction and pollination are therefore relatively independent from unpollinated fruits.

Although both CPPU and pollination can induce fruit set in horticultural crops, the mechanisms by which they achieve this display some differences. Histological analysis revealed smaller cell size and higher cell density in CPPU-induced fruits compared with pollinated fruits, indicating that CPPU-induced fruit set mainly promotes cell division, whereas pollination-induced fruit set results in more cell expansion accompanied by cell division (Supplementary Data Fig. S2). This was corroborated by changes in phytohormone levels. Auxin promotes cell division and expansion and GA promotes cell expansion, while ABA increases cell senescence [30–32]. Endogenous auxin, GA, and ABA levels in CPPU-induced fruits show similar trends to those in pollinated fruits, but the degree of change is significantly different. CPPU promotes and maintains GA accumulation, but to significantly lower levels than in pollinated fruits; this may account for the smaller cell size in CPPU-induced fruits compared with pollinated fruits. However, this study, as well as others in tomato, kiwifruit, and grape, confirms that an increase and maintenance of the GA level are required for CPPU-induced fruit set [19, 21, 26]. Differences in gene transcript levels also suggest differences between CPPU treatment and pollination. In pear, tomato, cucumber, and melon, numerous key genes in phytohormone pathways are significantly changed after spraying with CPPU, while pollination causes changes in genes associated with c+ell proliferation.

Hormones have long since been a hot topic in the study of parthenocarpy [6, 7]. The fruit set process is accompanied by changes in phytohormone levels and transcription levels of related genes, as well as the interaction between phytohormones [3]. CK plays a key role in various developmental processes, including seed germination, meristem activation and identity, and leaf senescence [23, 24, 33]. CPPU, an active CK, is a major regulator for inducing parthenocarpy. Its mechanism has been explored in tomato, cucumber, and kiwifruit, and completion of its induction of fruit set depends on interaction with the auxin or GA pathways [11, 21, 34]. *KAO* and *GA20ox* encode key enzymes for GA synthesis, and their expression level directly affects the content of bioactive GAs. During numerous developmental processes, GA and CK typically elicit opposite effects [35], while both GA and CK have synergistic effects on fruit set and development [7]. In melon, CPPU induces an increase in GA level and upregulation of biosynthetic gene expression, maintaining these during fruit set. GO analysis of genes specifically expressed during CPPU-induced fruit set revealed that enrichment of CK signal transduction genes is accompanied by changes in GA-responsive and signal transduction genes. Exogenous application of the GA biosynthesis inhibitor PAC suppresses CPPU-induced fruit set (Fig. 4). Knockdown of *CmRR2*, a class-B *RR* highly expressed during fruit set, reduces CPPU-induced *CmGA20ox1* expression. Direct binding of *CmRR2* to the promoter of *CmGA20ox1* was confirmed by luciferase (LUC) and Y1H assay results (Fig. 5). Taken together, these results indicate that CPPU may directly induce the expression of GA biosynthesis genes through the CK signaling pathway in melon, promoting GA biosynthesis to complete fruit set (Fig. 5f). CK and GA are therefore important for fruit set in melon. Unfortunately, the *CmRR2* transgenic material was sterile, and the self-crossing fruits showed abnormal development, like the intermediate state. After harvesting the seeds of the transgenic lines, the seeds of the mature fruits were deformed and small (Supplementary Data Fig. S9). Recent studies have also shown that type-B ARR regulates sexual reproduction in *Arabidopsis* [46]. We speculated that the altered RR gene expression affected melon reproduction, so that sufficient seeds could not be obtained for phenotypic analysis of fruit setting. The function of *CmRR2* in fruit set should be studied using gene editing technology [36]. Spraying different concentrations of GA induced fruit set in melon but could not achieve 100% fruit set (Supplementary Data Fig. S7); we speculated that CPPU-induced fruit set was largely but not completely dependent on GA production. Interestingly, CPPU induces fruit set independently of the GA pathway in pear [22]. CPPU increases the content of auxin by acting on the promoter of *PbYUCCA4* through *PbRR9* in the CK pathway; it also inhibits the expression of the ABA biosynthesis gene *PbNECD6*, thus reducing the content of ABA. We showed that the content of IAA is significantly upregulated and the content of ABA is significantly downregulated after treatment with CPPU. GO enrichment results indicated that terms related to auxin and ABA are also enriched during fruit set. This is consistent with results obtained from tomato, pear, and cucumber and suggests that CPPU-induced fruit set relies on increased auxin level and decreased ABA level, whereas the opposite occurs in unpollinated ovaries (Fig. 2). CPPU-induced fruit set relies on direct or indirect communication with other phytohormones (Fig. 5f), although the mechanism of action differs among species. Although preliminary and limited, our results provide a reference for creating parthenogenetic germplasm resources.

## Materials and methods

### Plant material and growth conditions

In the present work, we used the melon ivf05 varieties provided by Dr H. Wang from the Institute of Vegetables and Flower (IVF), Chinese Academy of Agricultural Sciences. All materials were planted at the Agricultural Hi-tech Industry Zone, Jimo, Shandong (36°56′N, 120°21′E) in a plastic green house in 2020, 2021 and 2022.

### Pharmacological treatments

Bisexual flower ovaries at the first node of the lateral branch were selected for experimental analysis. At −1 DAA, flowers were artificially emasculated and the ovaries were soaked on the day of anthesis. Four groups of samples were collected with different treatments: no pollination; artificial pollination; CPPU (Yuanye, S18139); and CPPU supplemented with PAC (Yuanye, S80628). CPPU (50 mg) and PAC (100 mg) were dissolved in 10 ml ethanol and diluted in water containing 0.02% Tween to the working concentration. Only the fruit part was retained when sampling organs and was collected from −2 to −3 DAA.

### Fruit set rate and size determination

The fruits/ovaries collected after 10 DAA for different treatments were used for the calculation of fruit set rate and the measurement of fruit size. Fruit set rate (%) was calculated as follows: number of enlarged fruits divided by total fruit number. Fruit size was measured using ImageJ on photographs made using a camera. Fruit width was the length of the widest longitudinal section of the fruit. Fruit length was the distance from the stalk to the umbilicus in the longitudinal section.

### Paraffin sectioning

The samples to be observed were fixed in fixatives (formaldehyde, acetic acid, ethanol) and evacuated and stored at 4°C. An ethanol/xylene series was used to dehydrate samples, which were further embedded in paraffin. Next, we dried the 10-μm sections after cutting, and stained them with safflower and fast green. The sections were examined with an Olympus CX22LED light microscope. We quantified cell size and density with ImageJ software (http://rsbweb.nih.gov/ij/) as described previously [37]. We confirmed our results by non-biased double-blinded analyses.

### Phytohormone analysis

Samples, ground in liquid nitrogen, were extracted using 90% aqueous methanol. Extracts were reconstituted with aqueous methanol and analyzed by liquid chromatography–tandem mass spectrometry (LC–MS/MS). Sample separation was performed on an ultra-high-performance liquid chromatography system (Shimadzu Nexera X2 LC-30 AD) with a C18 column (2.1 × 150 mm, 5 μm, 100 Å) and a 400 μl/min flow rate. We used 0.1% formic acid in water as mobile phase A and 0.1% formic acid in acetonitrile as mobile phase B. The gradient was 2–98% B in 10 minutes, 98–2% B in 0.1 minute, and 2% B for 2 minutes. An AB SCIEX 5500 Q-trap mass spectrometer was used for sample detection in positive ion mode. The parameters of MS analysis were as follows: source temperature at 500°C; ion spray volume at 5500v; ion source gas all at 45; and curtain gas at 30. The ion pairs to be measured were detected using MRM mode.

### Transcriptome sequencing

The total RNA of sample was extracted to construct the cDNA library, which was sequenced on the Illumina NextSeq platform (Annoroad Gene Technology Beijing Co. Ltd). Sequences of 150-bp paired ends were generated. Each group consisted of three replicates (Supplementary Data Table S1).

### Data processing

Using Hisat2 v2.2.1 with the default parameters, we mapped the clean reads to the melon reference genome (V4) [38]. For each gene expression, the FPKM value was calculated using StringTie v2.1.7 to estimate the gene expression level [39]. FPKM values of all melon genes were used for PCA and PCC analyses with the help of Prcomp and cor.test in R, respectively. The DEGs were screened using R package DEseq2 [40] by applying the thresholds of log_2_ (fold change) >1 and adjusted *P*-value <.05.

### Gene Ontology enrichment analyses

The functional categories of DEGs were obtained from GO enrichment analysis using R package clusterProfiler [41]. A corrected *P* value of <.05 was set as the significance cutoff.

### Dual luciferase assay

The p1305.4 plasmid containing the *CmRR2* coding sequence was used as the effector. To produce *pCmGA20ox1*-LUC double-reporter vector, the promoter sequence (2000 bp) of *CmGA20ox1* was introduced into the pGreen II 0800-LUC vector. The effector vector was transformed into *Agrobacterium tumefaciens* strain GV3101 and the reporter into GV3101 (pSoup-p19), both of which were co-infiltrated (volume ratio of 9:1) into the leaves of 4-week-old *N. benthamiana* with the empty vector as control [42]. Luciferase activities were determined from the leaf samples collected within 60 hours after injection using the Firefly Luciferase Reporter Gene Assay Kit (Beyotime, RG005) and recorded by a multi-mode and imaging reader (BioTek, Cytation 1) according to the manufacturer’s instructions. The LUC/REN ratio was calculated to determine the relative reporter gene expression level. Each sample contained three independent transformations. The primers used in this experiment are provided in Supplementary Data Table S3.

### ChIP-seq data analysis

We obtained the ARR10 ChIP-seq data from the NCBI Sequence Read Archive (http://www.ncbi.nlm.nih.gov/sra) [47] (accession numbers SRX732967 and SRX733461). In the Plant Chromatin State Database, computational analyses of protein binding were performed as described previously [48]. Using the UCSC Genome Browser, we retrieved the distribution of the mapped read abundance in the promoter regions of GA20ox1 in the Plant Chromatin State Database [48].

### Yeast one-hybrid assay

Yeast one-hybrid assays were performed following the user manual of the Matchmaker Gold Yeast One-Hybrid System kit (Clontech). The *CmRR2* coding sequence was cloned into pGADT7 to produce effectors associated with a GAL4-activation domain. The sequence of 250 bp near *CmRR2* binding site 1 and 135 bp near *CmRR2* binding site 2 were synthesized and introduced into the pAbAi (SacI and SalI) plasmid to produce corresponding reporters. Before transformation into Y1H Gold, the pAbAi-bait vectors were linearized. We selected the colonies using plates coated with selective synthetic dextrose medium (SD) free of uracil. Correct integration into Y1H Gold was verified through PCR. The bait yeast strains containing the AD prey were selected on SD/−Leu/AbA (200, 300, or 400 ng/ml) plates. All transformations and screenings include triplicates. The primers used for amplification are shown in Supplementary Data Table S3.

### *Agrobacterium*-mediated transformation

The translation method was modified based on previous studies [43]. The constructed *35S:CmRR2* (1305.4) vector was used for *Agrobacterium* transformation. Two days before transformation, we amplified the vector-carried *Agrobacterium* stock cells (EHA105) in liquid LB medium shaken at 200 rpm for 12 hours at 28°C. The LB medium contained 50 mg/l kanamycin and 50 mg/l rifampicin. *Agrobacterium*-mediated transformation: (4.43 g/l Murashige and Skoog, 0.5 g/l 2-Morpholinoethanesulphonic acid monohydrate, 2 mg/l 6-Benzylaminopurine, 80 mg/l Acetosyringone, 1 mg/l ABA, 30 g/l sucrose) to OD600 value 0.2. The seeds were sterilized. Twenty-four hours later the cotyledon of the germinated seeds was cut in half transversely, and we removed the hypocotyl and cut a U-shaped incision at the proximal end of the seed. Next, we transferred these treated explants to the *Agrobacterium* suspension followed by sonication using an ultrasonic cleaning instrument (SB-5200DTD). The explants were then transferred to a 20-ml medical syringe containing 15 ml of *Agrobacterium* suspension, and the plunger was slowly pulled so that the *Agrobacterium* could infiltrate into the explants under vacuum. Following this, the explants were transferred with filter paper and cultured in darkness for 3 days with solid medium B (4.43 g/L MS, 3.3 g/L Gelzan, 1 mg/l ABA, 0.5 g/l MES, 80 mg/l AS, 2 mg/l 6-BA, 150 mg/l dithiothreitol (DTT), 30 g/l sucrose). Next, the explant samples were rinsed with sterile water and transferred to solid medium C (4.43 g/l MS, 1 mg/l ABA, 3.3 g/l Gelzan, 200 mg/l Timentin, 30 g/l sucrose, 0.5 mg/l 6-BA, 2 mg/l Basta). Three to four weeks later, explants selected with PCR were transferred to the MS medium to root. The final rooted seedlings were transferred to soil to grow.

### RNA extraction and real-time qPCR

Total RNA was isolated using the Quick RNA Isolation Kit (Huayueyang, 0416GK) according to the manufacturer’s instructions. Reverse transcription was performed using GoScript™ Reverse Transcription Mix (Promega, A2790). The primers used for qPCRs were SP330/SP331 for *CmRR2* and SP342/SP343 for *CmGA20ox1*. The primers used to amplify SP58/SP59 for *ACTIN* were as described [42]. All primers are listed in Supplementary Data Table S3.

## Acknowledgements

We thank Professor Huaisong Wang for providing ivf05 seeds. We also thank Biorun Bioscience Co., Ltd (Wuhan) for Dr Jin Jie’s contributions to the genetic transformation system of melon. This work was supported by the National Natural Science Foundation of China (32225044 to Z.Z., 32130093 to Z.Z., 32102404 to S.C., 32002064 to Y.L.), This work was also supported by the Taishan Scholar Foundation of the People’s Government of Shandong Province and the Natural Science Foundation of Shandong Province (ZR2020QC157).

## Author contributions

Z.Z., S.C., and Y. Liu. designed the research. S.C., H.G., Y. Li., J.F., and H.W. performed the experiments and S.C. and Y. Liu. analyzed the data. B.L. revised the manuscript. Z.Z., S.C. and Y. Liu. wrote the manuscript. All authors participated in the research and approved the final manuscript.

## Data availability

Relevant data can be found within the paper and its supporting materials. All data of this study are available from the corresponding author upon reasonable request.

## Conflict of interest

All authors declare no conflict of interest.

## Supplementary data

Supplementary data is available at *Horticulture Research* online.

## Supplementary Material

Web_Material_uhad084
