# Supplementary material for: Gibberellin biosynthesis is required for CPPU-induced parthenocarpy in melon
Source: Hortic Res. 2023 May 3;10(6):uhad084. doi: 10.1093/hr/uhad084 (PMC10266944; doi:10.1093/hr/uhad084)
Supplement: Web_Material_uhad084 [file web_material_uhad084.zip › Supplemental figures(clean).docx]

**Supplementary Information**

**Gibberellin biosynthesis is required for CPPU-induced parthenocarpy in melon**

**Authors:** Yue Liu^1†^, Yang Li^1†^, Huixin Guo^†^, Bingsheng Lv^1^, Jing Feng^1^, Huihui Wang^1^, Zhonghua Zhang^1^* and Sen Chai^1^*

**Supporting Information**

**Figure S1.** Identification of optimal CPPU concentrations for induce fruit set in ivf05.

**Figure S2.** Histological observations of melon pericarps during fruit set.

**Figure S3.** Gibberellin (GA_1_, GA_3_, GA_7_) and Cytokinin (cZ, iP, DZ) content of ivf05 during fruit set stages.

**Figure S4.** The FPKM value boxplot for each sample gene.

**Figure S5.** Volcano plots of DEGs in the pairwise comparisons.

**Figure S6.** The number of up/down-regulated genes in CPPU-treated, pollinated and unpollinated fruits compared with mature ovary.

**Figure S7.** Different concentrations of gibberellin induced fruit set.

**Figure S8.** Expression pattern of melon type-B *RR* in various tissues.

**Figure S9.** Type B *RR* gene expression and fruit and seed phenotypes in RR2 transgenic lines.

**Figure S10.** Schematic diagram of GA synthetic gene promoters and their expression levels in transgenic lines.

**Figure S11.** Binding profile of CmRR2 to *CmGA20ox1* promotor.

**Figure S12.** Phylogenetic analysis of *GA20ox1*.

**Table S1.** Statistics of RNA-seq data.

**Table S2.** List of DEGs in the pairwise comparisons.

**Table S3.** List of primers.

**Table S4.** GO analysis of differentially expressed genes in fruit set.

**Table S5.** GO analysis of differentially expressed genes in unpollinated fruit.


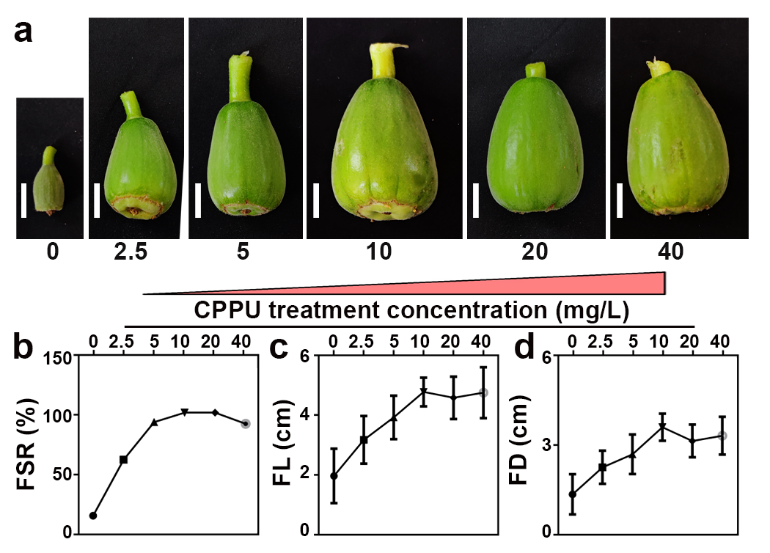


**Figure S1.** **Identification of optimal CPPU concentrations for induce fruit set in ivf05.** a Morphological observation of fruit set under different concentrations (0-40 mg/L) of CPPU treatment at 10 DPA. Scale bar = 1 cm. b-d Fruit set rate (FSR), fruit length (FL) and fruit diameter (FD) under different concentrations of CPPU treatment. Bars = means ± SEM (n = 10) of at least three independent trials.


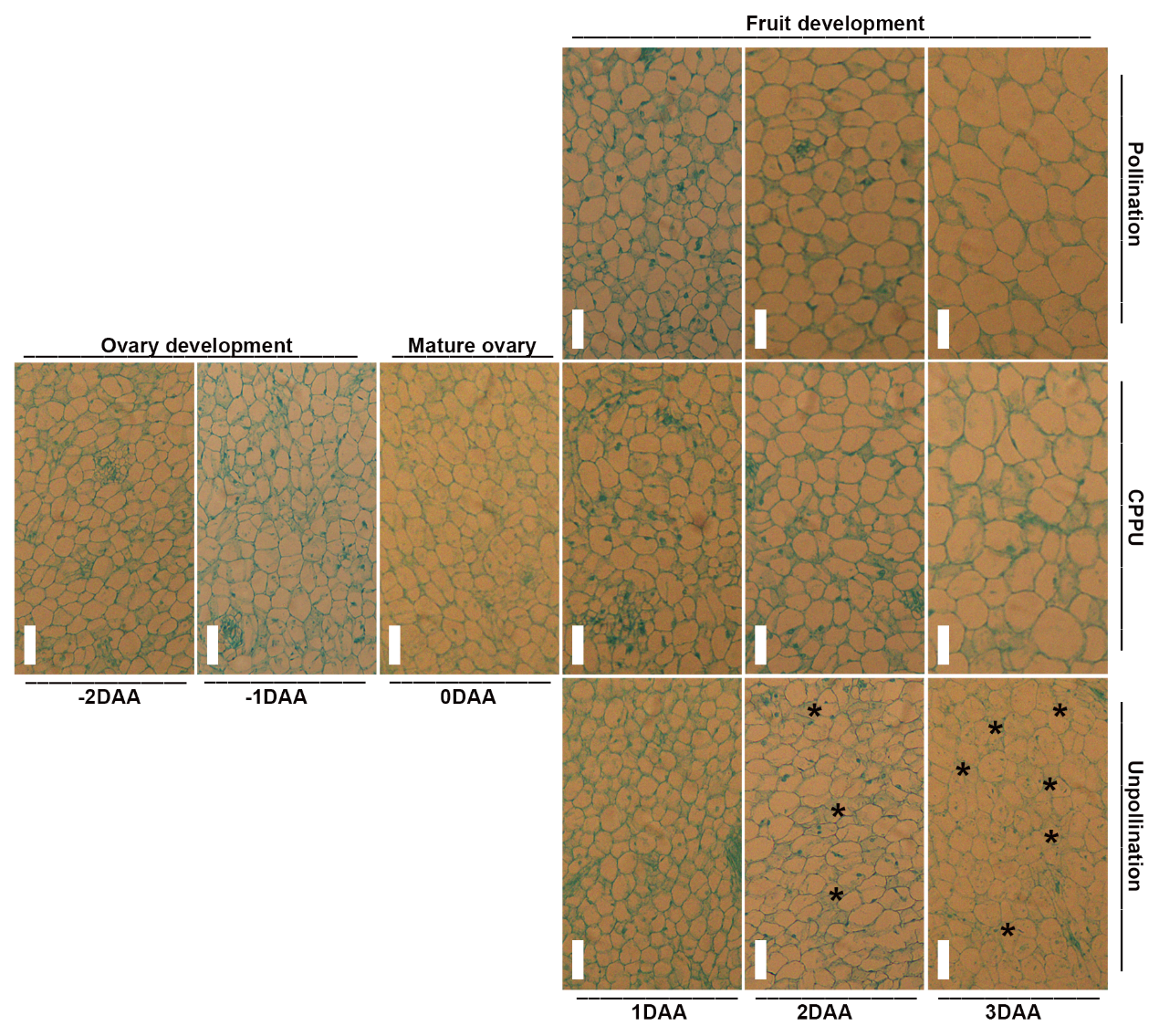


**Figure S2.** **Histological observations of melon pericarps during fruit setting.** Microscopic transverse sections of the pericarp of a melon fruit at fruit set, including ovary development, mature ovary, and fruit set in different treatments. Bars = 50 μm. The asterisks indicate abnormal cells.


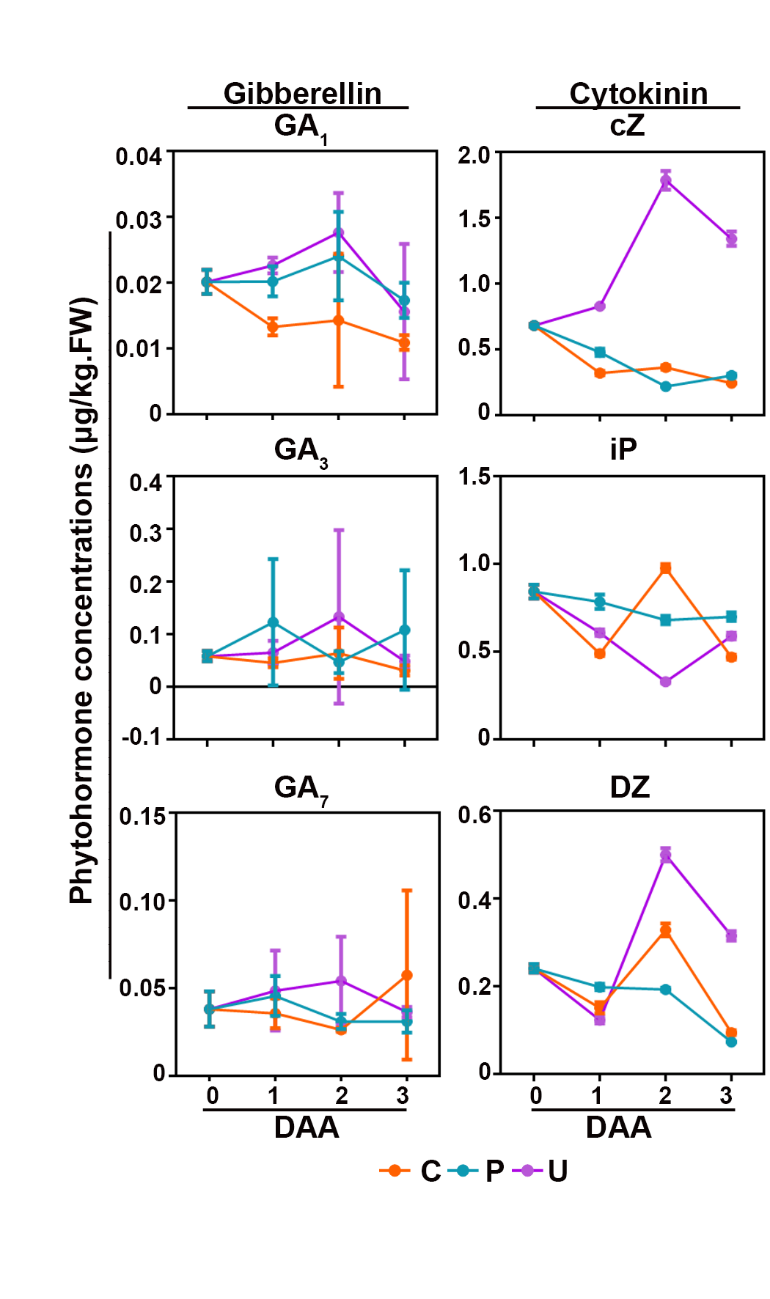


**Figure S3.** **Gibberellin** **(GA_1_, GA_3_, GA_7_) and Cytokinin (cZ, iP, DZ) content in the fruit of ivf05 during fruit set stages.** The concentrations of the Gibberellin (GA1, GA3, GA7) and Cytokinin (cZ, iP, DZ) were analyzed by LC-MS. C, CPPU-treated (orange); P, pollinated (blue); U, unpollinated (purple). The lines indicate the up or down trend of the hormone levels. Error bars indicate mean ± SD of at least three independent trials.


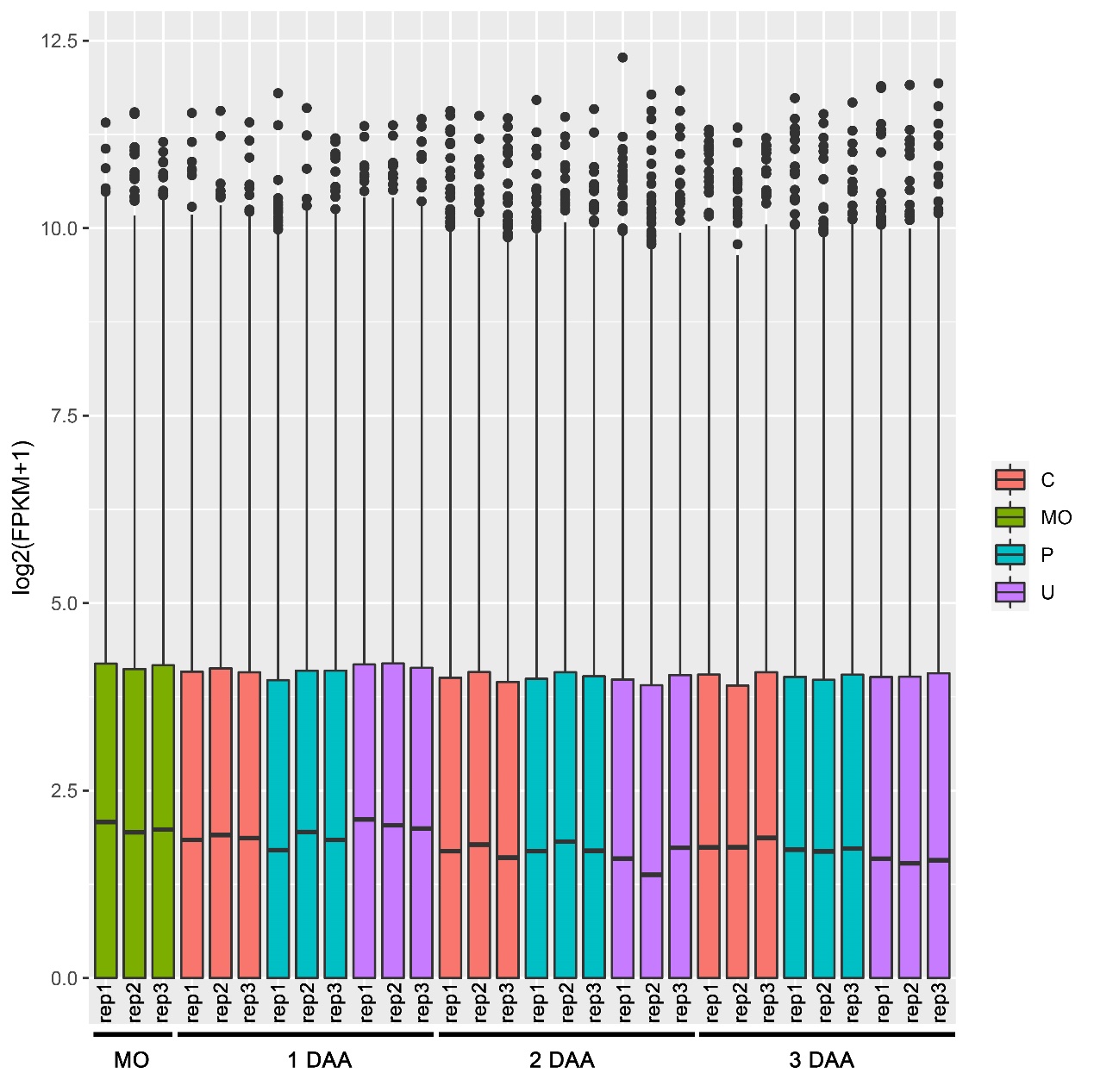


**Figure S4.** **The FPKM value boxplot for each sample gene.**


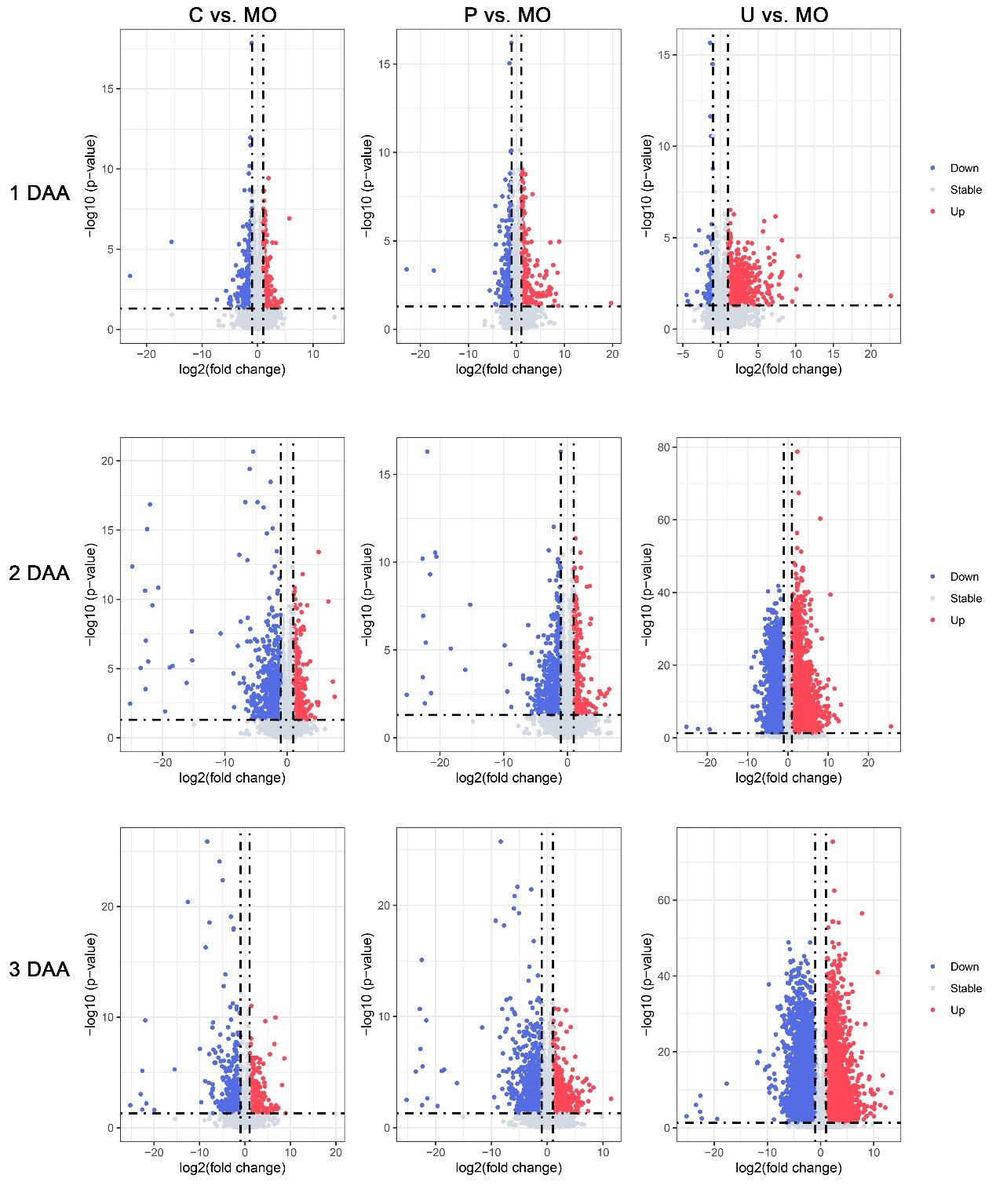


**Figure S5.** **Volcano plots of DEGs in the pairwise comparisons** (red dots represent up-regulated DEGs, blue dots represent down-regulated DEGs, gray dots represent genes that are not differentially expressed, the abscissa represents the fold change in gene expression in different samples, and the ordinate represents statistically significant differences in gene expression).


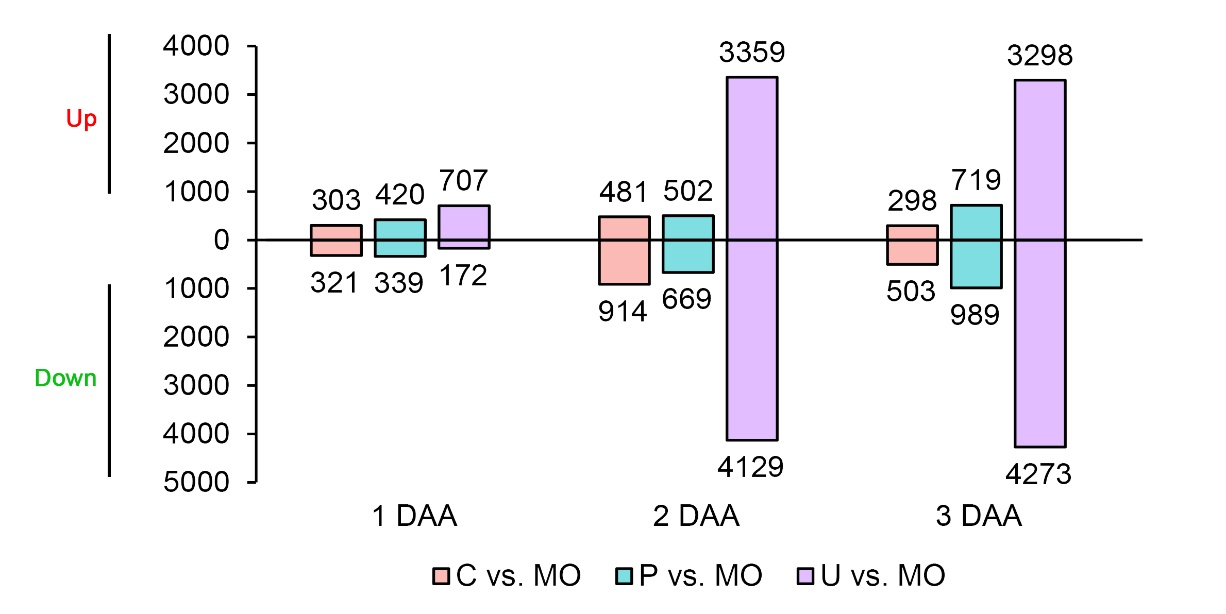


**Figure S6.** **The number of up/down-regulated genes in CPPU-treated, pollinated and unpollinated fruits compared with mature ovary.**


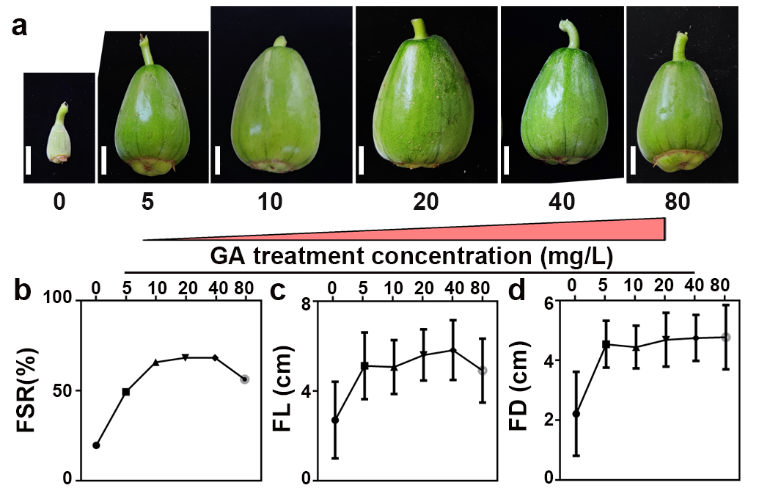


**Figure S7.** **Different concentrations of gibberellin induced fruit set.** a Morphological observation of fruit set under different concentrations (0-80 mg/L) of GA treatment at 10 DAA. Scale bars = 1 cm. b-d Fruit set rate (FSR), fruit length (FL) and fruit diameter (FD) under different concentrations of gibberellin treatment. Bars = means ± SEM (n = 10) of at least three independent trials.


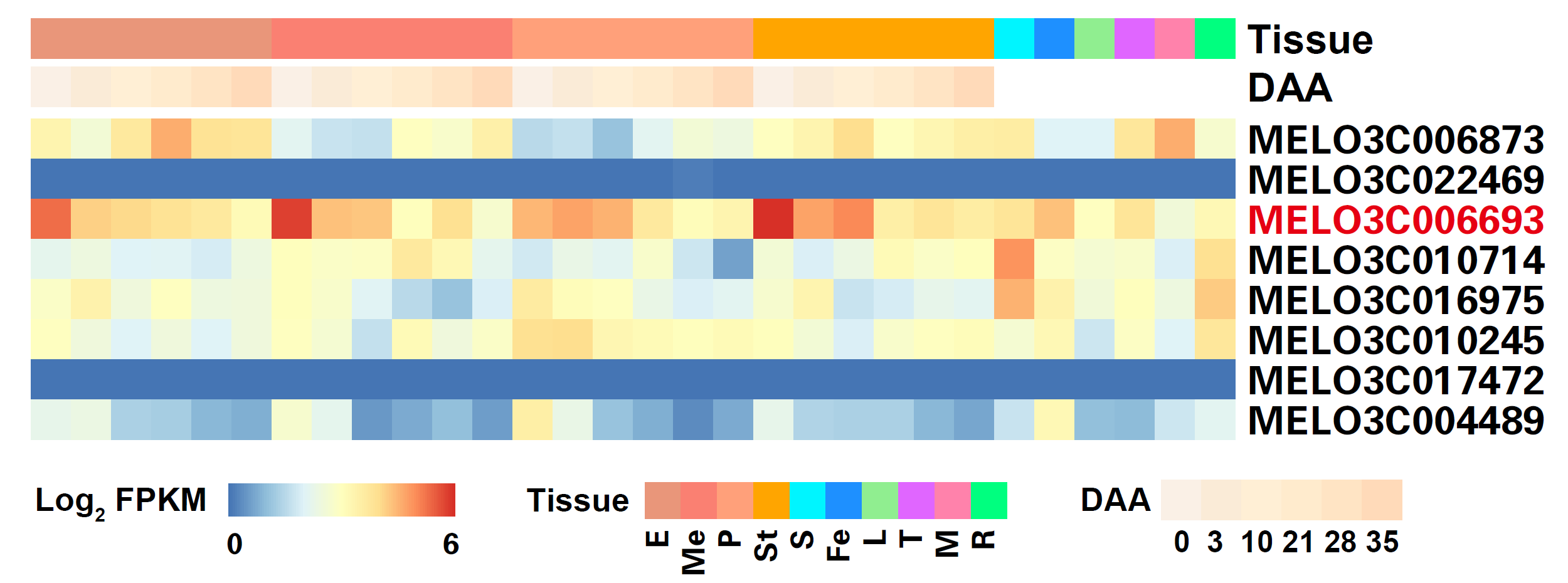


**Figure S8.** **Expression pattern of melon type-B *RR* in various tissues.** Heat map for type-B ARR gene expression in various melon tissues across development time. E, epidermis; Me, mesocarp; P, placenta; St, stalk; S, stem; Fe, female; L, leaf; T, tendril; M, R, root; DAA, day after anthesis.


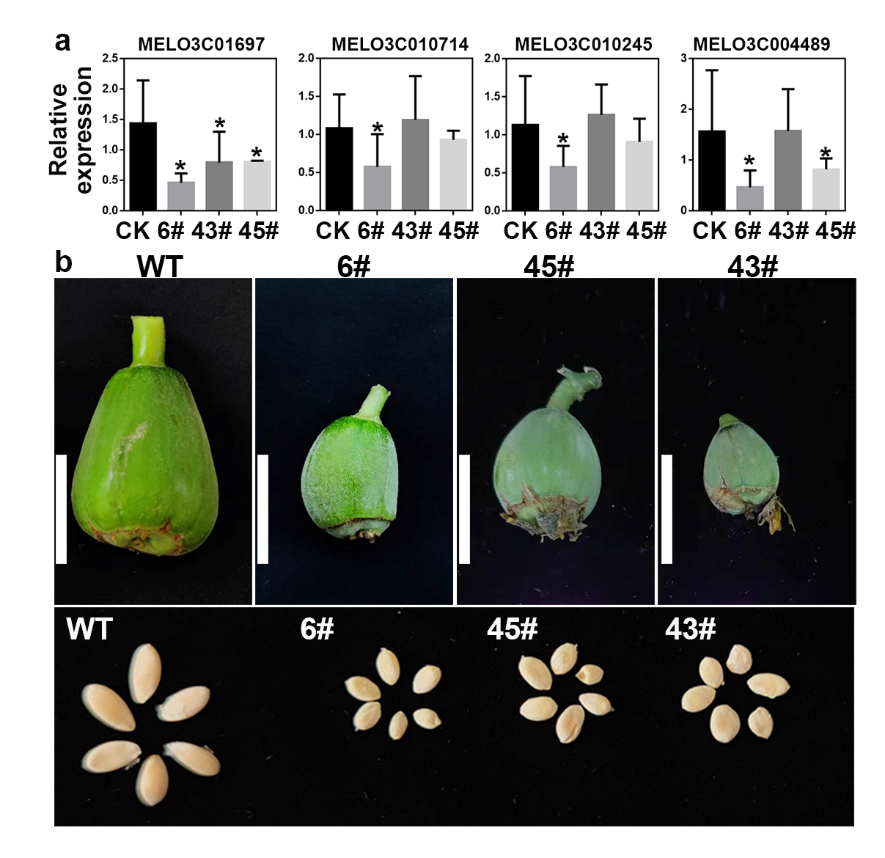


**Figure S9. Type B *RR* gene expression and fruit and seed phenotypes in RR2 transgenic lines**

a qRT-PCR analysis of type B *RR* gene (MELO3C016975, MELO3C010714, MELO3C010245 and MELO3C004489) in fruits of the wild type and different transgenic lines 6#, 43# and 45#. b Morphological observation of fruit development (10 DAA) and seeds in *RR2* transgenic lines (n=3, mean±SD, *t*-test: *, *P* < 0.5.). Scale bars = 2.5 cm.


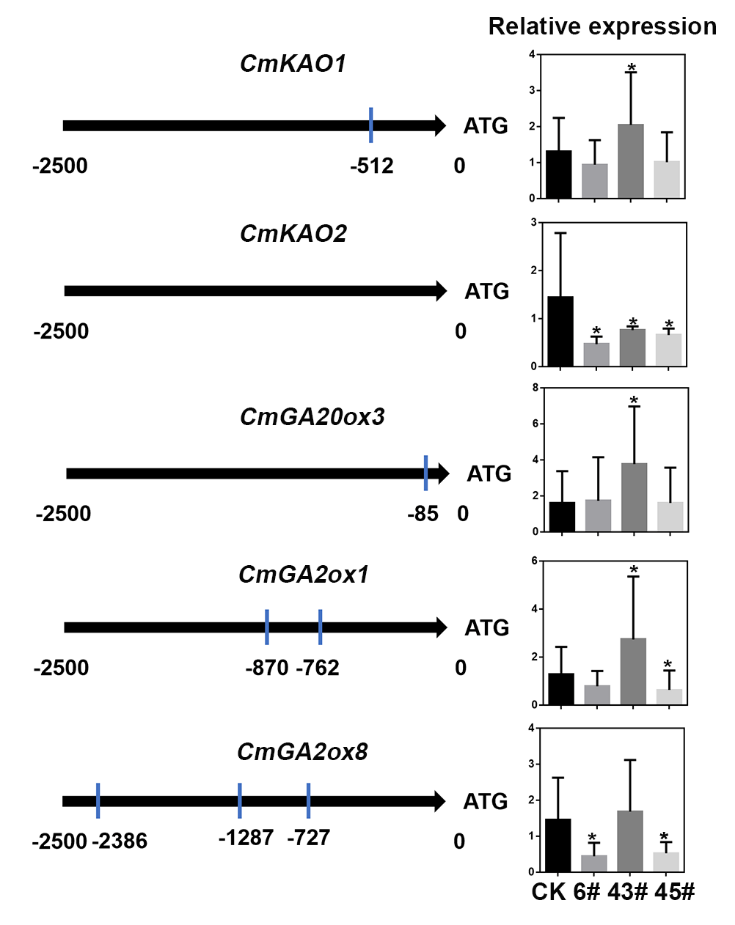


**Figure S10. Schematic diagram of GA synthetic gene promoters and their expression levels in *RR2* transgenic lines.** The blue lines indicate the class B RR-binding sites. qRT-PCR analysis of

*CmKAO1* (MELO3C016975), *CmKAO2* (MELO3C006237), *CmGA20ox3* (MELO3C030020), *CmGA2ox1* (MELO3C016152) and *CmGA2ox8* (MELO3C034481) in fruits of the wild type and different *RR2* transgenic lines 6#, 43# and 45# (n=3, mean±SD, *t*-test: *, *P* < 0.05.).

f

**
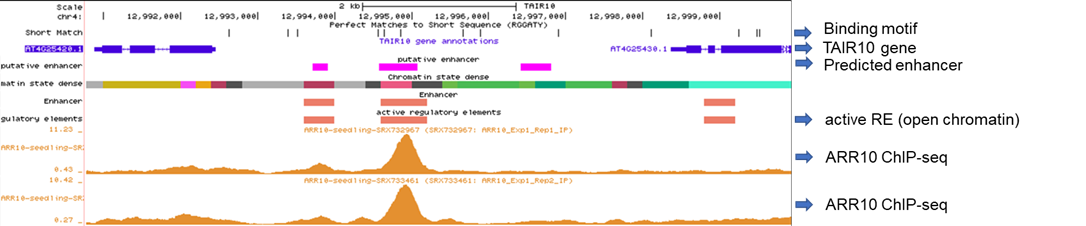
**

**Figure S11.** **Binding profile of ARR10 to *GA20ox1* promotor.** Genome browser view on location of *AtGA20ox1* (AT4G25420) and its upstream regions of Arabidopsis thaliana genome (TAIR10). The tracks from top to bottom are base position, position of AtARR10 binding motif, predicted enhancer, chromatin states, enhancer, activate regulatory elements (predicted based on open chromatin), AtARR10 ChIP-seq signals (SRA accession: SRX732967 and SRX733461). The RPM (reads per million mapped reads) value of ChIP-seq data is indicated on the left side of the track. The information of chromatin states and regulatory elements comes from PCSD database.


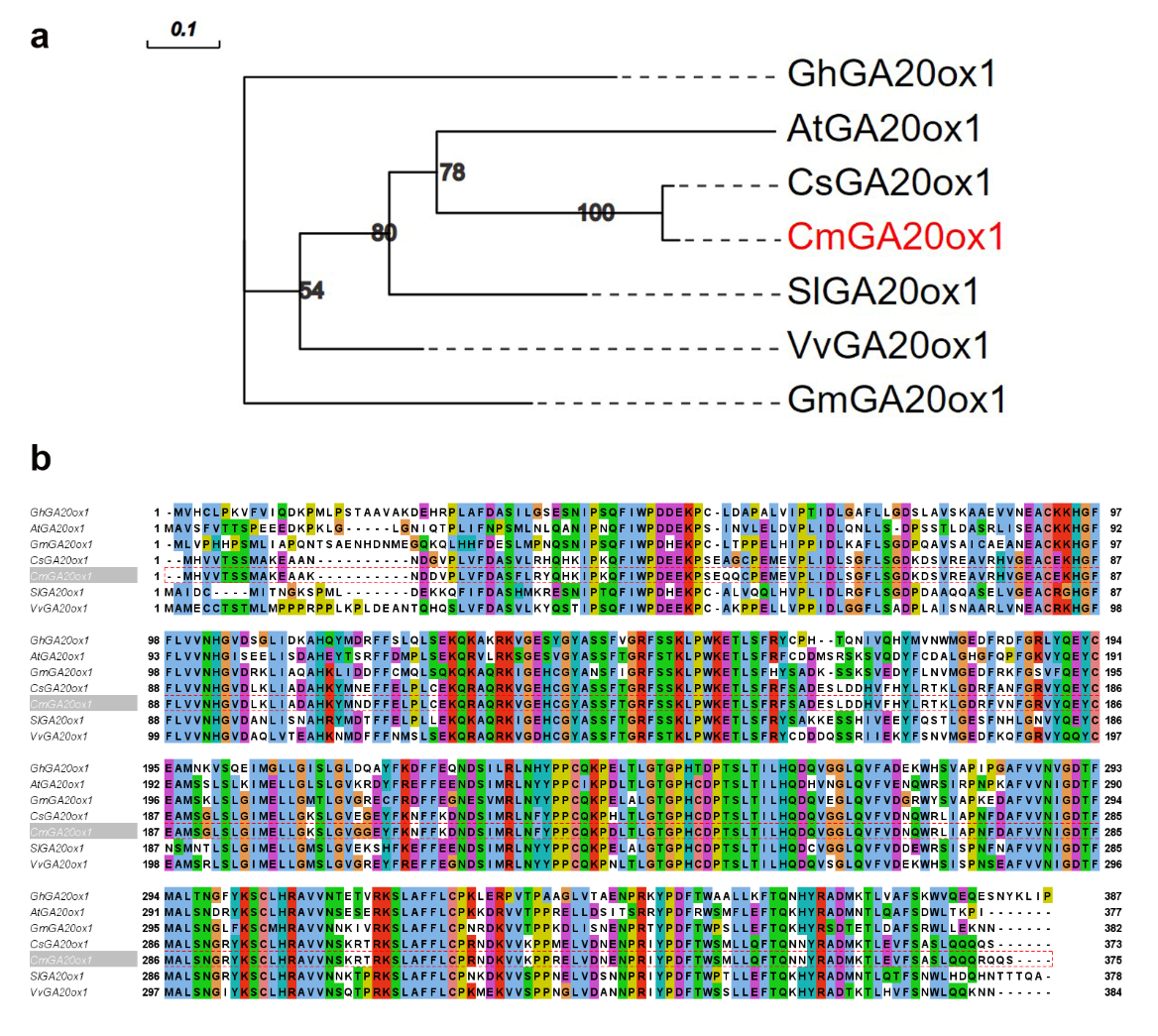


**Figure S12. Phylogenetic analysis of *GA20ox1*.** a Phylogenetic tree of GA20ox1 from different plant species. Numbers represent the percentage bootstrap values. b Multiple sequence alignment of the GA20ox1 from Arabidopsis, tomato, cucumber, grape, soybean and cotton. CmGA20ox1 is highlighted with a gray background and red dashed box. Gene accession numbers corresponding to GA20ox1 are as follows: *Arabidopsis thaliana*: AGW24332.1; *Solanum lycopersicum*: NP_001234070.1; *Cucumis sativus*: XP_004143646.1; *Vitis vinifera*: AGQ42623.1; *Glycine max*: XP_003521460.1; *Gossypium hirsutum*: XP_016745676.1
